# Obstructive sleep apnea and mental disorders: a bidirectional mendelian randomization study

**DOI:** 10.1186/s12888-024-05754-8

**Published:** 2024-04-23

**Authors:** Heming Liu, Xuemei Wang, Hu Feng, Shengze Zhou, Jinhua Pan, Changping Ouyang, Xiaobin Hu

**Affiliations:** Department of Epidemiology and Health Statistics, School of Public Health, Lanzhou University, No.199, Donggang West Road, Chengguan District, 730000 Lanzhou, Gansu Province China

**Keywords:** Mental disorders, Obstructive sleep apnea (OSA), Two-sample bidirectional mendelian randomization

## Abstract

**Background:**

Previous studies have reported associations between obstructive sleep apnea (OSA) and several mental disorders. However, further research is required to determine whether these associations are causal. Therefore, we evaluated the bidirectional causality between the genetic liability for OSA and nine mental disorders by using Mendelian randomization (MR).

**Method:**

We performed two-sample bidirectional MR of genetic variants for OSA and nine mental disorders. Summary statistics on OSA and the nine mental disorders were extracted from the FinnGen study and the Psychiatric Genomics Consortium. The primary analytical approach for estimating causal effects was the inverse-variance weighted (IVW), with the weighted median and MR Egger as complementary methods. The MR Egger intercept test, Cochran’s Q test, Rucker’s Q test, and the MR pleiotropy residual sum and outlier (MR-PRESSO) test were used for sensitivity analyses.

**Result:**

MR analyses showed that genetic liability for major depressive disorder (MDD) was associated with an increased risk of OSA (odds ratio [OR] per unit increase in the risk of MDD, 1.29; 95% CI, 1.11–1.49; *P* < 0.001). In addition, genetic liability for OSA may be associated with an increased risk of attention-deficit/hyperactivity disorder (ADHD) (OR = 1.26; 95% CI, 1.02–1.56; *p* = 0.032). There was no evidence that OSA is associated with other mental disorders.

**Conclusion:**

Our study indicated that genetic liability for MDD is associated with an increased risk of OSA without a bidirectional relationship. Additionally, there was suggestive evidence that genetic liability for OSA may have a causal effect on ADHD. These findings have implications for prevention and intervention strategies targeting OSA and ADHD. Further research is needed to investigate the biological mechanisms underlying our findings and the relationship between OSA and other mental disorders.

**Supplementary Information:**

The online version contains supplementary material available at 10.1186/s12888-024-05754-8.

## Background

Mental disorders are a significant global health concern and rank among the top 10 causes of burden worldwide. According to estimates, the number of mental disorder cases increased by 48.1% in 2019 compared to 1990, and the proportion of global disability-adjusted life years (DALYs) attributable to mental disorders increased from 2.3–9.4% [1]. However, the etiology of mental disorders remains unclear, and there is evidence to suggest that Obstructive sleep apnea (OSA) may be associated with a variety of mental disorders [2]. OSA is a condition caused by repeated episodes of upper airway collapse and obstruction during sleep associated with arousal from sleep with or without oxygen desaturation [3]. In the general population, the prevalence of OSA is approximately 3–7% in males and 2–5% in females [4]. Both mental disorders and OSA can significantly impact patients’ quality of life. It is crucial to establish a clear causal relationship between these conditions to inform effective prevention and treatment strategies.

In recent years, an increasing number of researches have been devoted to exploring the relationship between OSA and various mental disorders. Two cohort studies suggested that bipolar disorder (BD) and schizophrenia (SCZ) were associated with increased risks of OSA [5, 6]. A Japanese study has also found that preschoolers with autism spectrum disorder (ASD) are more likely to have obstructive sleep apnea than the general population [7]. Moreover, there is evidence of a potential bidirectional relationship between OSA and attention deficit hyperactivity disorder (ADHD), anxiety disorder (ANX), major depressive disorder (MDD), and Post-Traumatic Stress Disorder (PTSD) [8–11]. However, the ability of these studies to establish causality is insufficient, and even prospective observational studies may be subject to inherent confounding or selection bias. Currently, there are limited studies on this topic, and it remains unclear whether OSA is the cause or a downstream effect of mental disorders. Therefore, any potential causal relationship still requires further research.

In this case, the genetic epidemiological method of Mendelian randomization (MR) is a powerful tool to evaluate the causal relationship between OSA and mental disorders. MR uses single nucleotide polymorphisms (SNPs) as instrumental variables to estimate their effect on the outcome of interest, minimizing bias affecting observational epidemiological studies [12–14] and thus enhancing causal inference of exposure and outcome. Due to the random allocation of genetic variation during meiosis and the natural causal effect of genetic variation on phenotype, SNPs are independent of potential confounders, and therefore confounders and reverse causality bias can be minimized [15]. Two-sample MR refers to MR analysis using two independent samples from different studies or databases, which are typically collected from publicly available large-scale genome-wide association studies (GWAS), and has the advantage of increased statistical power [16]. Therefore, we conducted a two-sample bidirectional MR analysis using the latest GWAS to investigate the potential association between OSA and mental disorders.

## Method

### Study design

**Fig. 1:**
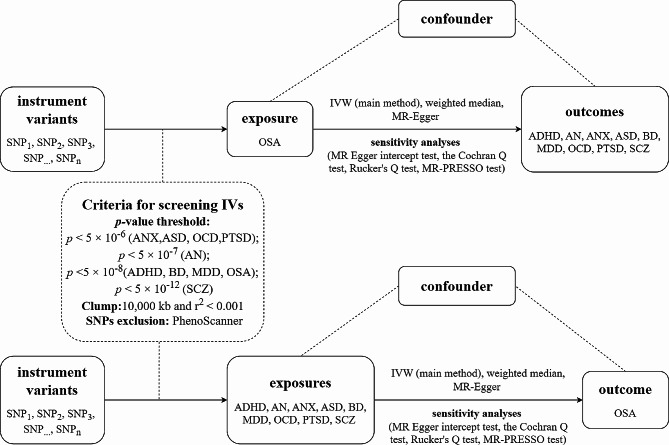
Workflow of a two-sample bidirectional MR study on obstructive sleep apnea and mental disorders.

A two-sample bidirectional MR was used to evaluate the potential causal association between obstructive sleep apnoea and nine mental disorders (Fig. 1). The MR design is based on three fundamental assumptions three basic assumptions: (1) genetic variants must be highly correlated with exposure; (2) genetic variants cannot be associated with any potential confounders; (3) genetic variants influence outcome solely through the exposure [15]. This study used summary-level data from publicly available GWAS. Ethical approval was obtained for all original studies.

### Data source

#### Obstructive sleep apnea

Summary statistics for OSA were obtained from the FinnGen database with 375,657 individuals of European ancestry. FinnGen is a large public-private partnership aimed at collecting and analyzing genomic and health data from 500,000 Finnish biobank participants [17]. The GWAS included 38,998 OSA cases and 336,659 controls. Diagnostic criteria for OSA cases were based on ICD codes (ICD-10: G47.3; ICD-9: 3472), derived from the Finnish National Hospital Discharge Registry and the Causes of Death Registry. More details on the OSA GWAS can be found at https://r9.risteys.finngen.fi/endpoints/G6_SLEEPAPNO/ and https://finngen.gitbook.io/documentation/methods/phewas/.

#### Mental disorders

We used summary statistics for 9 mental disorders from different studies [18–26], which were obtained from the Psychiatric Genomics Consortium (PGC). The mental disorders included in this study are ADHD, AN, ANX, autism spectrum disorder (ASD), BD, MDD, obsessive-compulsive disorder (OCD), PTSD, and SCZ. For consistency with the OSA data, genetic data of European ancestry were used for all nine mental disorders to avoid heterogeneity. Sample sizes for each GWAS study are listed below: ADHD(38,691 cases and 186,843 controls), AN(16,992 cases and 55,525 controls), ANX(7,016 cases and 14,745 controls), ASD(18,381 cases and 27,969 controls), BD(41,917 cases and 371,549 controls), MDD(246,363 cases and 561,190 controls), OCD(2,688 cases and 7,037 controls), PTSD(23,212 cases and 151,447 controls), SCZ(53,386 cases and 77,258 controls). A detailed description of the data sources can be found in Additional file 1.

### Selection of instrument variables

The number of instrumental variables (IVs) determines statistical power and the presence of confounding factors, so the appropriate number of IVs is needed. We used four different *p*-value thresholds, *p* < 5 × 10^− 6^ (ANX, ASD, OCD, PTSD), *p* < 5 × 10^− 7^ (AN), *p* < 5 × 10^− 8^ (ADHD, BD, MDD, OSA), and *p* < 5 × 10^− 12^ (SCZ), and without linkage disequilibrium (10,000 kilobase pairs apart and r^2^ < 0.001) to select SNPs as instrumental variables. The linkage disequilibrium (LD) reference was obtained from http://fileserve.mrcieu.ac.uk/ld/1kg.v3.tgz. After clumped SNPs for independence, PhenoScanner [27, 28] was used to assess previous associations with potential confounding traits. PhenoScanner is a curated database holding publicly available results from large-scale genome-wide association studies. To meet the assumptions of the MR design, we excluded SNPs that were strongly associated with other traits or diseases (*p* < 5 × 10^− 8^) to rule out possible pleiotropic effects. To evaluate the weak instrument bias, we calculated the instrument strength (F-statistic) for each IV according to the following formula: $F= \frac{{beta}^{2}}{{se}^{2}}$ (beta: the effect size of SNP on exposure; se: its corresponding standard error) [29]. We removed SNPs associated with exposure if they could not be matched to SNPs in the outcome. Details of the SNPs that were selected as IVs are shown in Additional files 2 and 3.

### Statistical analysis

In MR analysis, the inverse-variance weighted (IVW) model is the main method for assessing the bidirectional relationship between exposure and outcome. However, if there is horizontal pleiotropy and invalid instrument bias, IVW cannot provide unbiased estimates of causal effects [30, 31]. Therefore, we use two different MR methods that are relatively robust to horizontal pleiotropy, although at the cost of reduced statistical power [32]. First, causal effects are estimated using the weighted median method, in which only 50% of the SNPs need to be valid instruments [33]. Second, MR Egger estimates causal effects by setting an intercept that allows horizontal pleiotropy to be unbalanced or directed [31, 34]. In addition, we used several sensitivity analyses including the MR Egger intercept test, Cochran’s Q test, Rucker’s Q test and the MR pleiotropy residual sum and outlier (MR-PRESSO) test to determine the validity and robustness of the results. The MR Egger regression provides a test for directional pleiotropy through its intercept [35]. The Cochran’s Q test and Rucker’s Q test were performed to assess the heterogeneity, and if heterogeneity was present, outlier SNPs were excluded by observing the funnel plot or sorting RSSobs in MR-PRESSO and the MR analysis was repeated [36]. MR-PRESSO was used to detect pleiotropic bias, identify outliers, and obtain the corrected results by removing outliers [37].

Associations between genetic liability for OSA and risk of mental disorders were expressed as odds ratios (ORs) and their 95% confidence intervals (CIs). We indexed the strength of evidence against the no association by the exact *p*-value. A *p*-value less than 0.0056 (0.05/9) was considered to be statistically significant evidence for a causal association. A *p*-value below 0.05, but above the Bonferroni-corrected threshold, was considered suggestive evidence for a potential causal association. We used an online tool called mRnd (https://shiny.cnsgenomics.com/mRnd/) to calculate the statistical power of the MR analysis [38]. All statistical analyses were performed using R (version 4.3.1) with the packages “TwoSampleMr”, “MR-PRESSO” and “ieugwasr”.

## Results

### Genetically predicted obstructive sleep apnea on mental disorders

**Fig. 2:**
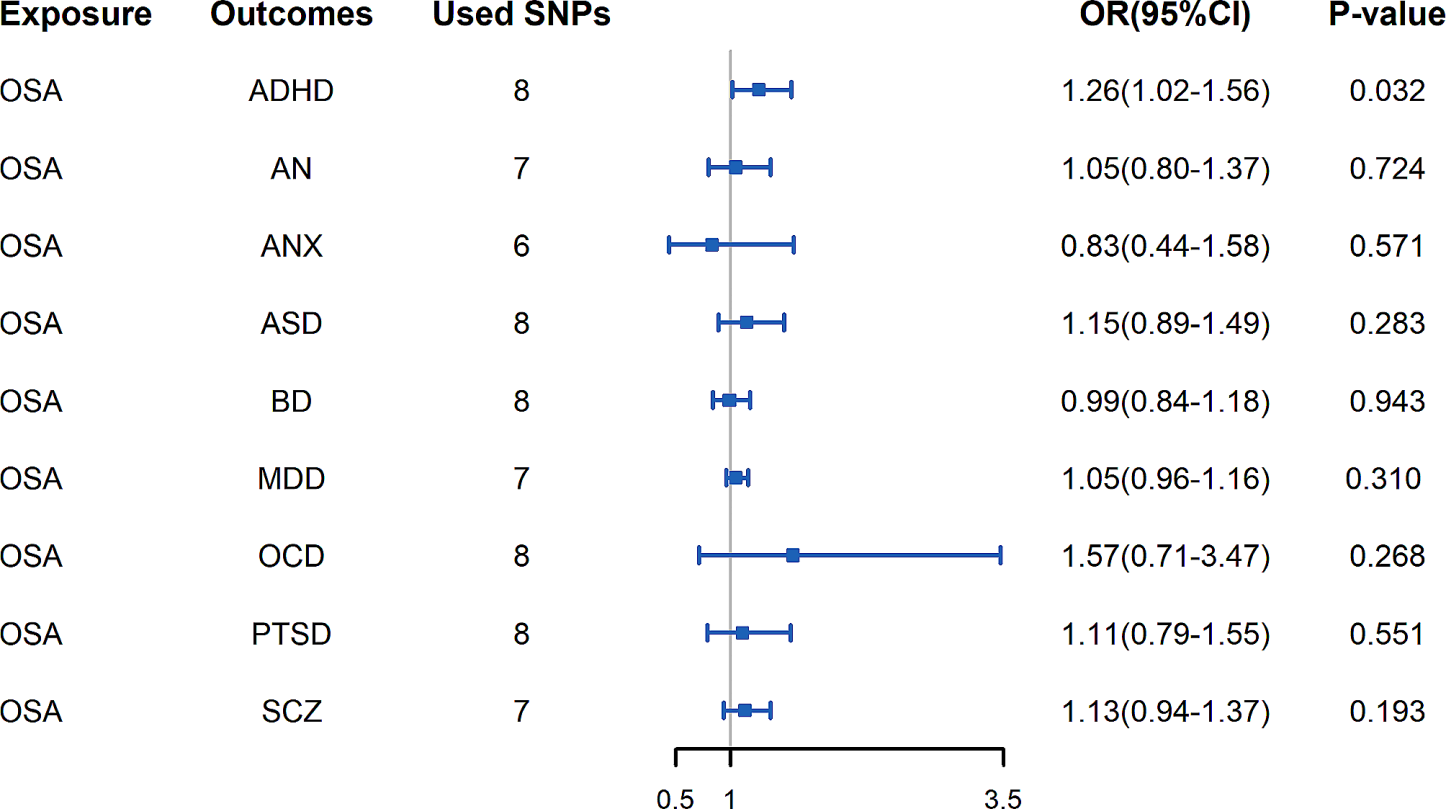
Associations between genetic liability for obstructive sleep apnea and risk of psychiatric disorders. For outcome phenotype SCZ, the result is shown after removing outliers due to heterogeneity.

The associations of OSA with 9 mental disorders are demonstrated in Fig. 2. We observed only a weak association between genetic liability for OSA and increased risk of ADHD (IVW, OR = 1.26; 95% CI, 1.02–1.56; *p* = 0.032), and there was no evidence that other mental disorders were associated with OSA. The results of the estimation using the weighted median and MR Egger methods, as well as information on statistical power, Q *P*-value and *P*_*intercept*_-value, are presented in Additional file 4. The F-statistic of each IV we selected for OSA was greater than 10, indicating a low probability of weak instrument bias [39]. The MR-Egger intercept analysis did not indicate horizontal pleiotropy. The Rucker’s Q test revealed possible heterogeneity of individual SNPs between the effect estimates of OSA and SCZ (Rucker’s Q = 13.964, Q *p*-value = 0.030). Therefore, we excluded one outlier SNP (rs59333125) and performed a second MR analysis, which showed no heterogeneity (Rucker’s Q = 6.670, Q *p*-value = 0.246). For the SNPs we used, MR-PRESSO did not detect any potential outliers.

### Genetically predicted mental disorders on obstructive sleep apnea

**Fig. 3:**
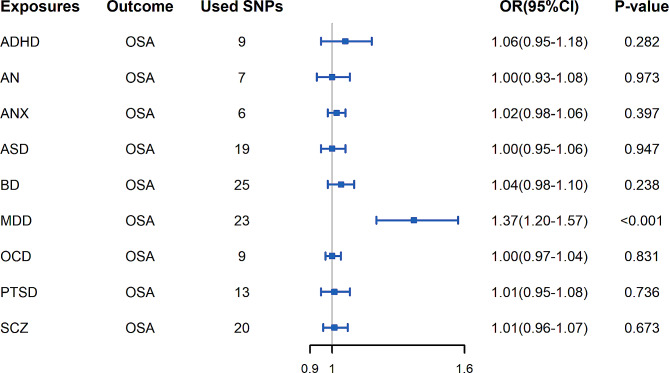
Associations between genetic liability for mental disorders and risk of obstructive sleep apnea. For exposure phenotype ASD, the result is shown after removing outliers with the MR-PRESSO test. For exposure phenotype ADHD, BD, MDD, PTSD and SCZ, the figure shows the results of the second MR analysis with outlier SNPs removed due to heterogeneity.

The associations of 9 mental disorders with OSA are demonstrated in Fig. 3. The genetic liability for MDD had an effect estimate consistent with an increased risk of OSA. However, Cochran’s Q test indicated the presence of heterogeneity (Cochran’s Q = 43.195, Q *p*-value = 0.009), we excluded two outlier SNPs (rs4141983, rs9529218) and performed a second MR analysis (IVW, OR = 1.37; 95%CI, 1.20–1.57; *p* < 0.001), which did not show heterogeneity (Cochran’s Q = 29.474, Q *p*-value = 0.132). The results of the estimation using the weighted median and MR Egger methods, as well as information on statistical power, Q *P*-value and *P*_*intercept*_-value, are presented in Additional file 5. The scatterplot, leave-one-out-sensitivity forest plot, and funnel plot of MR estimation results for MDD associated with OSA are provided in Additional file 6. F-statistic greater than 10 for each IV for the 9 mental disorders, indicating a small magnitude of weak instrument bias. MR-Egger intercept analysis did not identify any pleiotropic SNPs. And Cochran’s Q test suggested potential heterogeneity in ADHD (Cochran’s Q = 28.816, Q *p*-value = 0.001, BD (Cochran’s Q = 38.784, Q *p*-value = 0.039), PTSD (Cochran’s Q = 22.605, Q *p*-value = 0.047) and SCZ (Cochran’s Q = 38.169, Q *p*-value = 0.012), so we excluded outlier SNPs for ADHD (rs7844069, rs2025286), BD (rs10994415), PTSD (rs1268149), and SCZ (rs145071536, rs16851048) and performed a second MR analysis, which showed no heterogeneity (Additional file 5). One outlier SNP in ASD (rs28729902) was excluded using the MR-PRESSO test. After correction for possible outliers, the causal effect estimates for ADHD, ASD, BD, PTSD and SCZ were still not statistically significant.

## Discussion

In our study, we performed a bidirectional MR analysis based on several large genetic populations to assess the association between genetic liability for OSA and the risk of mental disorders. Our results found possible genetic evidence that OSA was associated with an increased risk of ADHD. In the opposite direction, genetic liability for MDD was associated with an increased risk of OSA.

Our study provides suggestive evidence of a possible association between OSA and ADHD based on larger populations. Although few observational studies have conclusively confirmed the potential causal relationship between OSA and ADHD, there are still evidences that support our findings. A meta-analysis showed that children with Sleep-disordered breathing (SDB) are at increased risk of presenting with ADHD symptoms such as inattention and hyperactivity [40]. OSA is usually accompanied by decreased oxygen saturation and sleep disruption. These symptoms may affect brain development, which in turn affects cognitive function and leads to poor concentration [41, 42]. From a pathophysiological perspective, inflammatory cytokines (C-reactive protein and interleukin-6) are elevated in children with SDB, which may lead to cognitive dysfunction [43–45]. In addition, several studies have shown that adenotonsillectomy (a treatment for OSA) can improve ADHD symptoms and cognitive problems [8, 40, 46–48]. However, the relationship between OSA and ADHD may be reciprocal rather than in the traditional one-way relationship, although a reverse relationship was not observed in our study. Symptoms of ADHD overlap with a diagnosis of OSA, and attentional deficits have been reported in up to 95% of OSA patients [49]. A case-control study found that 28 out of 30 ADHD patients had comorbid sleep disorders, 15 of whom had OSA [50]. In summary, the relationship between OSA and ADHD is complex and needs to be further explored in future studies.

Our study found that MDD was associated with an increased risk of OSA incidence, which is consistent with a recent MR study [51]. However, observational studies similar to ours are limited. Only one population-based longitudinal study suggested that a bidirectional link between MDD and OSA exists. This study, which included 27,073 depressed patients and 135,365 controls, demonstrated that having depression was associated with an increased risk of future OSA (HR = 2.30; 95%CI, 2.11–2.50) [10]. More observational studies consider OSA as a risk factor for MDD [52–55], so the relationship between MDD and OSA seems to be reciprocal as well. Two systematic reviews showed that both the prevalence of MDD among patients with OSA and the prevalence of OSA among patients with MDD were higher than in the general population [56, 57]. This may be due to a partial overlap of symptoms and diagnostic criteria or common underlying mechanisms between MDD and OSA [58–60]. However, the following pathophysiological mechanisms support our derived unidirectional association between MDD and OSA. First, increased inflammatory cytokines in depressed patients may lead to neurological damage and altered circadian rhythms, which may increase the risk of OSA and exacerbate the symptoms of OSA [61, 62]. Second, patients with depressive disorders are usually associated with central nervous system 5-HT dysfunction, with decreased plasma tryptophan (a 5-HT precursor), decreased 5-HT metabolites in cerebrospinal fluid, and decreased 5-HT_1_ receptor binding [63]. The 5-HT reduction affects dilator muscles of the upper airway, narrowing the size of the upper airway, which may contribute to the incidence of OSA [64]. Third, some sleep medications and benzodiazepines are used to treat depression, and their tranquillizing effects may decrease the muscle tone of upper airway dilator muscles, thereby increasing the risk of OSA [65–67]. In conclusion, the relationship between MDD and OSA has not been clarified, and further research into pathogenesis is needed to provide effective and feasible treatments.

The main strength of this study is the use of two-sample bidirectional MR to assess the relationship between OSA and mental disorders, minimizing confounders and reverse causality present in observational studies. Moreover, we limited the population of the GWAS study to Europeans to minimize heterogeneity arising from ethnic differences. In addition, the IVs we used were extracted from the most recent GWAS with a large sample size, and the likelihood of weak instrument bias was minimal and only related to the exposures we were focused on.

However, there are several limitations to this study. First, both OSA and the nine mental disorders are binary exposures, and we could not know whether there would be selective bias due to underdiagnosis [29], as well as exclusive restriction bias due to the possibility that genetic variants would influence outcomes via continuous risk factors [68]. In addition, due to the lack of individual-level data from the GWAS study, we were unable to know whether the severity of the disease and medication would cause potential bias. Second, we used different thresholds for screening IVs, but this may have led to inconsistencies in the reliability of the results. Fewer IVs, while greatly reducing the potential for pleiotropy, reduced statistical power. Third, the biological roles and mechanisms of SNPs are not currently fully understood [69], and the presence of horizontal pleiotropy cannot be completely ruled out, although we performed sensitivity analyses using various methods to rule out horizontal pleiotropy. Fourth, MR estimates reflect the cumulative effects of exposure over individuals’ lifetimes, which are likely to be stronger than in observational studies and clinical trials. However, it is difficult to determine the relationship between OSA and mental disorders using randomized clinical trials due to the limitations of ethical rules and the uncertainty of etiology, and more observational studies are needed in the future to validate our findings. Finally, limiting the ancestry of the population to Europeans reduced the bias due to population stratification, but also limited the extrapolation of the results to other races.

## Conclusion

Our study provided genetic evidence that MDD is associated with an increased risk of OSA without a bidirectional relationship. In addition, we also found genetic evidence that OSA is a potential causal risk factor for ADHD. We did not find a causal relationship between OSA and AN, ANX, ASD, BD, OCD, PTSD, and SCZ. Clinically, these findings contribute to the identification, treatment, and prevention of OSA and ADHD in patients. Further investigation is required to better understand the biological mechanisms underlying the relationship between OSA and other mental disorders. This will aid clinicians in providing more effective treatment for these conditions.

### Electronic supplementary material

Below is the link to the electronic supplementary material.

Supplementary Material 1

Supplementary Material 2

Supplementary Material 3

Supplementary Material 4

Supplementary Material 5

Supplementary Material 6

## Data Availability

All data used in this study are obtained from open access databases or published manuscripts. FinnGen: https://r9.risteys.finngen.fi/endpoints/G6_SLEEPAPNO. https://storage.googleapis.com/finngen-public-data-r9/summary_stats/finngen_R9_G6_SLEEPAPNO.gz. PGC: https://pgc.unc.edu/for-researchers/download-results.

## Abbreviations

ADHD: Attention-deficit/hyperactivity disorder
AN: Anorexia nervosa
ANX: Anxiety disorder
ASD: Autism spectrum disorder
BD: Bipolar disorder
CI: Confidence interval
GWAS: Genome-wide association studies
IVs: Instrumental variables
IVW: Inverse variance weighted
LD: Linkage disequilibrium
MDD: Major depressive disorder
MR: Mendelian randomization
MR-PRESSO: Mendelian randomization pleiotropy residual sum and outlier test
OCD: Obsessive-compulsive disorder
OR: Odds ratio
OSA: Obstructive sleep apnea
PGC: Psychiatric Genomics Consortium
PTSD: Post-traumatic stress disorder
SCZ: Schizophrenia
SDB: Sleep-disordered breathing
SNP: Single nucleotide polymorphism
