# Supplementary material for: Obstructive sleep apnea and mental disorders: a bidirectional mendelian randomization study
Source: BMC Psychiatry. 2024 Apr 23;24:304. doi: 10.1186/s12888-024-05754-8 (PMC11040841; doi:10.1186/s12888-024-05754-8)
Supplement: Supplementary file 1 — Supplementary Material 1 [file 12888_2024_5754_MOESM1_ESM.doc]

**Additional file 1. Detailed information on GWAS summary statistics.**

| **Data source** | **Phenotypes** | **PMID** | **Population** | **Sample size** | | **Download address** |
| --- | --- | --- | --- | --- | --- | --- |
|  |  |  |  | **Cases** | **Controls** |  |
| Finngen | OSA | NA | European | 38,998 | 336,659 | https://storage.googleapis.com/finngen-public-data-r9/summary_stats/finngen_R9_G6_SLEEPAPNO.gz |
| PGC | ADHD | 36702997 | European | 38,691 | 186,843 | https://figshare.com/articles/dataset/adhd2022/22564390 |
| PGC | AN | 31308545 | European | 16,992 | 55,525 | https://figshare.com/articles/dataset/an2019/14671980 |
| PGC | ANX | 26754954 | European | 7,016 | 14,745 | https://figshare.com/articles/dataset/anx2016/14842689 |
| PGC | ASD | 30804558 | European | 18,381 | 27,969 | https://figshare.com/articles/dataset/asd2019/14671989 |
| PGC | BD | 34002096 | European | 41,917 | 371,549 | https://figshare.com/articles/dataset/PGC3_bipolar_disorder_GWAS_summary_statistics/14102594 |
| PGC | MDD | 30718901 | European | 246,363 | 561,190 | https://datashare.ed.ac.uk/handle/10283/3203 |
| PGC | OCD | 28761083 | European | 2,688 | 7,037 | https://figshare.com/articles/dataset/ocd2018/14672103 |
| PGC | PTSD | 31594949 | European | 23,212 | 151,447 | https://figshare.com/articles/dataset/ptsd2019/14672133 |
| PGC | SCZ | 35396580 | European | 53,386 | 77,258 | https://figshare.com/articles/dataset/scz2022/19426775 |

ADHD, attention-deficit/hyperactivity disorder; AN, Anorexia nervosa; ANX, anxiety disorder; ASD, autism spectrum disorder; BD, bipolar disorder; MDD, major depressive disorder; OCD, obsessive-compulsive disorder; OSA, obstructive sleep apnea; PGC, Psychiatric Genomics Consortium; PTSD, post-traumatic stress disorder; SCZ, schizophrenia
