# Supplementary material for: Obstructive sleep apnea and mental disorders: a bidirectional mendelian randomization study
Source: BMC Psychiatry. 2024 Apr 23;24:304. doi: 10.1186/s12888-024-05754-8 (PMC11040841; doi:10.1186/s12888-024-05754-8)
Supplement: Supplementary file 2 — Supplementary Material 2 [file 12888_2024_5754_MOESM2_ESM.doc]

**Additional file 2. Information on instrumental variables for obstructive sleep apnea.**

| **SNP** | **CHR** | **Position** | **Effect allele** | **Other allele** | **EAF** | **Beta** | **SE** | ***P*-value** | **F-statistic** |
| --- | --- | --- | --- | --- | --- | --- | --- | --- | --- |
| rs10507084 | 12 | 97359374 | T | C | 0.179 | 0.065 | 0.010 | 8.23E-11 | 42.20 |
| rs113955098 ^b,c^ | 10 | 18302297 | A | G | 0.067 | -0.099 | 0.016 | 3.38E-10 | 39.44 |
| rs114106239 ^b^ | 3 | 132779050 | T | C | 0.037 | -0.121 | 0.021 | 1.19E-08 | 32.51 |
| rs13114985 | 4 | 47302092 | G | T | 0.338 | 0.046 | 0.008 | 2.01E-08 | 31.48 |
| rs2016950 | 12 | 107586546 | T | C | 0.158 | -0.059 | 0.011 | 4.13E-08 | 30.09 |
| rs59333125 ^a^ | 12 | 56632124 | C | A | 0.081 | -0.082 | 0.014 | 1.32E-08 | 32.30 |
| rs679880 | 9 | 76652155 | A | G | 0.745 | 0.05 | 0.009 | 2.53E-08 | 31.04 |
| rs76229479 | 2 | 102507453 | C | A | 0.099 | -0.078 | 0.013 | 2.64E-09 | 35.44 |

^a^ This SNP cannot be extracted from the outcome of anorexia nervosa.

^b^ Two SNPs cannot be extracted from the outcome of anxiety disorder.

^c^ This SNP cannot be extracted from the outcome of major depressive disorder.

CHR, chromosome; EAF, effect allele frequency; SE, standard error; SNP, single nucleotide polymorphism.
