# Supplementary material for: Obstructive sleep apnea and mental disorders: a bidirectional mendelian randomization study
Source: BMC Psychiatry. 2024 Apr 23;24:304. doi: 10.1186/s12888-024-05754-8 (PMC11040841; doi:10.1186/s12888-024-05754-8)
Supplement: Supplementary file 3 — Supplementary Material 3 [file 12888_2024_5754_MOESM3_ESM.doc]

**Additional file 3. Information on instrumental variables for mental disorders used in the bidirectional Mendelian randomization.**

| **Phenotypes** | **SNP** | **CHR** | **Position** | **Effect allele** | **Other allele** | **EAF** | **Beta** | **SE** | ***P*-value** | **F-statistic** |
| --- | --- | --- | --- | --- | --- | --- | --- | --- | --- | --- |
| ADHD | rs11255890 | 10 | 8784773 | C | A | 0.389 | 0.053 | 0.010 | 4.14E-08 | 29.85 |
| ADHD | rs114142727 | 3 | 87015142 | C | G | 0.988 | 0.251 | 0.040 | 5.13E-10 | 38.67 |
| ADHD | rs115111850 | 3 | 43651029 | A | G | 0.944 | -0.115 | 0.020 | 1.71E-08 | 31.72 |
| ADHD | rs1162202 | 16 | 61966703 | C | T | 0.630 | 0.061 | 0.010 | 1.92E-09 | 36.23 |
| ADHD | rs17576773 | 4 | 112217523 | C | T | 0.888 | 0.096 | 0.015 | 1.63E-10 | 40.67 |
| ADHD | rs2025286 | 6 | 70858701 | A | C | 0.553 | -0.055 | 0.009 | 4.00E-09 | 34.85 |
| ADHD | rs2886697 | 3 | 20724204 | G | A | 0.634 | 0.059 | 0.010 | 7.90E-10 | 37.51 |
| ADHD | rs6537401 | 4 | 147099654 | G | A | 0.660 | -0.057 | 0.010 | 1.40E-08 | 32.27 |
| ADHD | rs76284431 | 14 | 98690923 | T | A | 0.847 | -0.082 | 0.013 | 1.19E-09 | 37.08 |
| ADHD | rs76857496 | 18 | 5871800 | C | A | 0.87 | 0.080 | 0.014 | 1.24E-08 | 32.66 |
| ADHD | rs7844069 | 8 | 93277087 | T | G | 0.428 | 0.055 | 0.010 | 6.74E-09 | 33.76 |
| AN | rs11615526 | 12 | 62216026 | G | A | NA | 0.090 | 0.017 | 1.52E-07 | 27.56 |
| AN | rs13125932 | 4 | 58150175 | C | T | NA | -0.072 | 0.013 | 5.85E-08 | 29.27 |
| AN | rs1539725 | 1 | 193153682 | T | C | NA | -0.073 | 0.014 | 1.26E-07 | 27.88 |
| AN | rs2287348 | 2 | 54039813 | C | T | NA | -0.104 | 0.018 | 5.62E-09 | 34.02 |
| AN | rs2821359 | 1 | 200004523 | T | C | NA | -0.082 | 0.016 | 3.76E-07 | 25.94 |
| AN | rs6789500 | 3 | 20764696 | T | C | NA | 0.091 | 0.018 | 2.55E-07 | 26.62 |
| AN | rs9874207 | 3 | 71019750 | T | C | NA | -0.081 | 0.015 | 2.05E-08 | 31.43 |
| ANX | rs1709393 | 3 | 101699154 | T | C | 0.579 | -0.151 | 0.027 | 1.65E-08 | 31.94 |
| ANX | rs2146346 | 6 | 11167111 | A | G | 0.587 | 0.144 | 0.030 | 1.38E-06 | 23.38 |
| ANX | rs2753188 | 6 | 47941339 | A | G | 0.727 | 0.160 | 0.033 | 1.42E-06 | 23.31 |
| ANX | rs28373923 | 16 | 88815473 | A | G | 0.068 | 0.419 | 0.092 | 4.56E-06 | 21.00 |
| ANX | rs58990403 | 11 | 116509339 | A | G | 0.792 | -0.185 | 0.039 | 2.16E-06 | 22.41 |
| ANX | rs739315 | 22 | 25617602 | A | G | 0.569 | -0.154 | 0.033 | 3.03E-06 | 21.83 |
| ASD | rs10110094 | 8 | 131472047 | A | G | NA | 0.091 | 0.019 | 2.05E-06 | 22.55 |
| ASD | rs11185408 | 1 | 104792257 | A | G | NA | -0.069 | 0.014 | 6.98E-07 | 24.78 |
| ASD | rs144911765 | 21 | 37255329 | T | C | NA | -0.190 | 0.040 | 2.36E-06 | 22.25 |
| ASD | rs2224274 | 20 | 14760747 | T | C | NA | 0.071 | 0.014 | 2.86E-07 | 26.47 |
| ASD | rs2391769 | 1 | 96978961 | A | G | NA | -0.077 | 0.015 | 1.14E-07 | 28.13 |
| ASD | rs28729902 | 9 | 76179384 | A | G | NA | -0.084 | 0.018 | 2.35E-06 | 22.22 |
| ASD | rs292441 | 18 | 55872558 | A | G | NA | -0.072 | 0.015 | 1.12E-06 | 23.67 |
| ASD | rs35404050 | 12 | 73196902 | T | C | NA | 0.084 | 0.018 | 1.61E-06 | 22.94 |
| ASD | rs45595836 | 10 | 16691399 | T | C | NA | 0.139 | 0.027 | 3.13E-07 | 26.11 |
| ASD | rs4750990 | 10 | 130488026 | T | C | NA | -0.068 | 0.014 | 1.37E-06 | 23.32 |
| ASD | rs6692705 | 1 | 193502609 | A | G | NA | 0.066 | 0.014 | 3.26E-06 | 21.65 |
| ASD | rs76397219 | 8 | 60390318 | A | G | NA | -0.140 | 0.030 | 3.57E-06 | 21.44 |
| ASD | rs77691144 | 13 | 66970212 | T | C | NA | -0.207 | 0.044 | 1.91E-06 | 22.73 |
| ASD | rs7783557 | 7 | 71646872 | T | C | NA | 0.067 | 0.015 | 4.36E-06 | 21.06 |
| ASD | rs78058104 | 15 | 93953737 | A | G | NA | 0.188 | 0.040 | 2.22E-06 | 22.4 |
| ASD | rs78653484 | 1 | 147183927 | T | C | NA | -0.176 | 0.039 | 4.68E-06 | 20.97 |
| ASD | rs78827416 | 10 | 72749037 | A | G | NA | 0.131 | 0.027 | 9.00E-07 | 24.07 |
| ASD | rs79940520 | 3 | 191838169 | A | G | NA | -0.095 | 0.021 | 4.26E-06 | 21.24 |
| ASD | rs910805 | 20 | 21248116 | A | G | NA | -0.096 | 0.016 | 2.04E-09 | 35.77 |
| ASD | rs9389208 | 6 | 135035609 | T | C | NA | 0.067 | 0.014 | 3.12E-06 | 21.78 |
| BD | rs10255167 | 7 | 140676153 | G | A | 0.215 | -0.066 | 0.012 | 1.60E-08 | 31.66 |
| BD | rs10737496 | 1 | 163745389 | C | T | 0.467 | 0.054 | 0.009 | 7.17E-09 | 33.25 |
| BD | rs10791849 | 11 | 65890597 | T | A | 0.819 | 0.069 | 0.012 | 9.89E-09 | 32.89 |
| BD | rs10866641 | 5 | 169289206 | T | C | 0.578 | 0.063 | 0.009 | 2.79E-11 | 44.35 |
| BD | rs10994415 | 10 | 62322034 | T | C | 0.912 | -0.118 | 0.017 | 1.14E-11 | 46.07 |
| BD | rs115694474 | 3 | 70488788 | T | A | 0.812 | 0.066 | 0.012 | 2.35E-08 | 30.95 |
| BD | rs11764361 | 7 | 105043229 | A | G | 0.671 | 0.061 | 0.010 | 3.47E-09 | 34.97 |
| BD | rs12575685 | 11 | 70517927 | G | A | 0.674 | -0.065 | 0.010 | 1.24E-10 | 41.67 |
| BD | rs17183814 | 2 | 166152389 | G | A | 0.933 | 0.103 | 0.019 | 2.68E-08 | 30.94 |
| BD | rs2011302 | 2 | 193738336 | T | A | 0.621 | -0.053 | 0.010 | 4.25E-08 | 29.75 |
| BD | rs237460 | 20 | 48033127 | C | T | 0.563 | -0.055 | 0.009 | 4.25E-09 | 34.61 |
| BD | rs28455634 | 16 | 9230816 | G | A | 0.626 | 0.063 | 0.010 | 2.63E-10 | 40.23 |
| BD | rs28565152 | 5 | 7542911 | G | A | 0.743 | -0.067 | 0.011 | 1.96E-09 | 35.89 |
| BD | rs2953928 | 8 | 34152492 | G | A | 0.941 | -0.116 | 0.020 | 6.25E-09 | 33.7 |
| BD | rs35958438 | 15 | 38973793 | G | A | 0.782 | 0.064 | 0.012 | 3.83E-08 | 30.11 |
| BD | rs4447398 | 15 | 42904904 | A | C | 0.137 | 0.082 | 0.014 | 2.61E-09 | 35.48 |
| BD | rs4619651 | 2 | 97416153 | G | A | 0.691 | 0.066 | 0.010 | 4.78E-11 | 42.83 |
| BD | rs5758064 | 22 | 41153879 | T | C | 0.524 | 0.052 | 0.009 | 2.01E-08 | 31.75 |
| BD | rs61554907 | 17 | 38220432 | G | T | 0.887 | -0.087 | 0.015 | 1.64E-08 | 31.77 |
| BD | rs62489493 | 8 | 9763581 | C | G | 0.856 | -0.090 | 0.014 | 2.64E-11 | 44.14 |
| BD | rs62581014 | 9 | 141066490 | C | T | 0.620 | -0.065 | 0.012 | 2.77E-08 | 31.05 |
| BD | rs6946056 | 7 | 131870597 | A | C | 0.382 | -0.053 | 0.010 | 3.66E-08 | 30.08 |
| BD | rs6954854 | 7 | 21492589 | G | A | 0.436 | 0.058 | 0.009 | 5.94E-10 | 38.46 |
| BD | rs696366 | 3 | 107757060 | C | A | 0.558 | 0.052 | 0.009 | 4.46E-08 | 30.13 |
| BD | rs7707252 | 5 | 78840610 | A | G | 0.716 | -0.057 | 0.010 | 3.64E-08 | 30.24 |
| BD | rs9834970 | 3 | 36856030 | T | C | 0.487 | -0.083 | 0.009 | 6.63E-19 | 79.65 |
| MDD | rs1021363 | 10 | 104851081 | A | G | 0.357 | 0.030 | 0.005 | 2.29E-11 | 44.44 |
| MDD | rs10501696 | 11 | 89014994 | A | G | 0.505 | 0.030 | 0.004 | 2.89E-11 | 44.95 |
| MDD | rs10913112 | 1 | 175944692 | T | C | 0.378 | -0.026 | 0.005 | 4.53E-09 | 33.90 |
| MDD | rs12919291 | 16 | 13706573 | C | G | 0.188 | 0.033 | 0.006 | 3.09E-09 | 35.35 |
| MDD | rs12967143 | 18 | 55431781 | C | G | 0.701 | -0.035 | 0.005 | 2.53E-13 | 53.88 |
| MDD | rs150346963 | 7 | 117985545 | T | C | 0.412 | 0.028 | 0.004 | 1.16E-10 | 41.37 |
| MDD | rs1931388 | 9 | 11203149 | A | G | 0.596 | 0.030 | 0.004 | 1.68E-11 | 44.95 |
| MDD | rs1950829 | 14 | 41628734 | A | G | 0.483 | 0.030 | 0.004 | 4.74E-12 | 47.71 |
| MDD | rs2214123 | 6 | 66290108 | A | G | 0.353 | 0.026 | 0.005 | 8.56E-09 | 33.64 |
| MDD | rs2418449 | 9 | 116969080 | T | C | 0.719 | 0.028 | 0.005 | 4.25E-09 | 34.27 |
| MDD | rs28541419 | 15 | 88402647 | C | G | 0.769 | 0.029 | 0.005 | 1.76E-08 | 31.53 |
| MDD | rs354155 | 1 | 49209604 | C | G | 0.092 | -0.045 | 0.008 | 1.75E-09 | 35.84 |
| MDD | rs3807865 | 7 | 12210776 | A | G | 0.411 | 0.031 | 0.004 | 1.09E-12 | 49.64 |
| MDD | rs4141983 | 1 | 17795514 | T | C | 0.674 | 0.026 | 0.005 | 9.69E-09 | 32.94 |
| MDD | rs508502 | 13 | 80347384 | T | C | 0.299 | -0.026 | 0.005 | 3.56E-08 | 30.25 |
| MDD | rs59082935 | 7 | 38685268 | T | C | 0.134 | 0.036 | 0.007 | 3.07E-08 | 30.25 |
| MDD | rs62535714 | 9 | 37182658 | A | G | 0.164 | 0.034 | 0.006 | 4.69E-09 | 34.16 |
| MDD | rs6656912 | 1 | 66617988 | T | C | 0.427 | -0.025 | 0.004 | 6.50E-09 | 34.35 |
| MDD | rs7152906 | 14 | 74658837 | T | C | 0.480 | -0.026 | 0.004 | 1.87E-09 | 36.00 |
| MDD | rs7241572 | 18 | 79820712 | A | G | 0.205 | 0.032 | 0.005 | 2.43E-09 | 35.78 |
| MDD | rs7551758 | 1 | 51808406 | T | G | 0.467 | -0.028 | 0.004 | 5.11E-11 | 43.31 |
| MDD | rs76954012 | 3 | 116258395 | A | T | 0.093 | 0.041 | 0.007 | 2.41E-08 | 31.00 |
| MDD | rs7725715 | 5 | 165060549 | A | G | 0.534 | 0.029 | 0.004 | 1.61E-11 | 45.48 |
| MDD | rs9364755 | 6 | 164696296 | A | G | 0.774 | -0.028 | 0.005 | 3.49E-08 | 30.79 |
| MDD | rs9529218 | 13 | 31215916 | T | C | 0.203 | -0.034 | 0.005 | 2.23E-10 | 39.64 |
| OCD | rs1030757 | 4 | 93697153 | A | C | NA | -0.165 | 0.034 | 1.09E-06 | 23.78 |
| OCD | rs117310268 | 18 | 19675267 | T | C | NA | 0.449 | 0.097 | 3.31E-06 | 21.62 |
| OCD | rs12568997 | 1 | 88867185 | A | G | NA | -0.293 | 0.058 | 4.23E-07 | 25.57 |
| OCD | rs4733767 | 8 | 128581578 | A | G | NA | 0.194 | 0.039 | 7.10E-07 | 24.62 |
| OCD | rs639560 | 15 | 42381240 | T | C | NA | -0.416 | 0.088 | 2.23E-06 | 22.38 |
| OCD | rs72781967 | 10 | 5622426 | T | C | NA | -0.167 | 0.035 | 2.43E-06 | 22.27 |
| OCD | rs72783425 | 16 | 14148431 | A | C | NA | 0.340 | 0.073 | 3.53E-06 | 21.48 |
| OCD | rs77885126 | 18 | 58420429 | T | C | NA | -0.603 | 0.131 | 4.38E-06 | 21.09 |
| OCD | rs9952159 | 18 | 3660801 | T | C | NA | 0.182 | 0.040 | 4.21E-06 | 21.19 |
| PTSD | rs117405401 | 12 | 131769084 | A | G | NA | 0.256 | 0.055 | 3.69E-06 | 21.41 |
| PTSD | rs1268149 | 6 | 109040325 | A | G | NA | 0.380 | 0.083 | 4.53E-06 | 21.02 |
| PTSD | rs12706983 | 7 | 131337495 | T | G | NA | -0.090 | 0.019 | 3.01E-06 | 21.78 |
| PTSD | rs139591016 | 4 | 99816170 | T | C | NA | -0.249 | 0.053 | 2.86E-06 | 21.89 |
| PTSD | rs140928208 | 5 | 15586134 | A | G | NA | 0.447 | 0.093 | 1.46E-06 | 23.19 |
| PTSD | rs1444764 | 3 | 123929043 | A | G | NA | 0.078 | 0.016 | 1.90E-06 | 22.68 |
| PTSD | rs149509653 | 11 | 118558425 | A | G | NA | 0.090 | 0.019 | 1.74E-06 | 22.93 |
| PTSD | rs17108326 | 14 | 72628507 | A | G | NA | 0.106 | 0.021 | 3.91E-07 | 25.72 |
| PTSD | rs2163050 | 2 | 41826738 | A | G | NA | 0.100 | 0.021 | 2.12E-06 | 22.46 |
| PTSD | rs36127550 | 12 | 133780309 | T | G | NA | -0.103 | 0.020 | 4.63E-07 | 25.34 |
| PTSD | rs73154700 | 22 | 31266823 | A | G | NA | 0.126 | 0.027 | 3.45E-06 | 21.46 |
| PTSD | rs763753 | 6 | 162168506 | A | G | NA | -0.114 | 0.022 | 2.43E-07 | 26.61 |
| PTSD | rs77537694 | 11 | 132725161 | A | G | NA | 0.130 | 0.028 | 3.25E-06 | 21.62 |
| PTSD | rs78608260 | 19 | 53637559 | A | G | NA | -0.321 | 0.069 | 3.71E-06 | 21.45 |
| SCZ | rs10035564 | 5 | 45252500 | A | G | 0.650 | -0.067 | 0.009 | 4.38E-13 | 52.72 |
| SCZ | rs11587347 | 1 | 239198959 | C | G | 0.895 | -0.104 | 0.015 | 1.53E-12 | 49.95 |
| SCZ | rs12129573 | 1 | 73768366 | C | A | 0.616 | -0.078 | 0.009 | 2.28E-18 | 76.41 |
| SCZ | rs12285419 | 11 | 46343189 | C | A | 0.800 | -0.085 | 0.011 | 1.05E-14 | 59.58 |
| SCZ | rs12293670 | 11 | 124612932 | A | G | 0.678 | 0.070 | 0.009 | 1.56E-14 | 58.72 |
| SCZ | rs145071536 | 1 | 243793012 | T | C | 0.799 | -0.085 | 0.012 | 1.62E-12 | 50.29 |
| SCZ | rs16851048 | 1 | 177276006 | T | C | 0.792 | -0.074 | 0.011 | 4.15E-12 | 48.47 |
| SCZ | rs2238057 | 12 | 2384005 | T | G | 0.568 | -0.084 | 0.009 | 8.50E-22 | 92.12 |
| SCZ | rs2332700 | 14 | 72417326 | C | G | 0.258 | 0.075 | 0.010 | 3.88E-14 | 57.54 |
| SCZ | rs2514218 | 11 | 113392994 | C | T | 0.668 | 0.070 | 0.009 | 1.35E-14 | 58.72 |
| SCZ | rs35351411 | 15 | 61872197 | A | C | 0.439 | -0.064 | 0.009 | 2.21E-13 | 53.28 |
| SCZ | rs4766428 | 12 | 110723245 | C | T | 0.539 | -0.075 | 0.009 | 3.93E-17 | 71.02 |
| SCZ | rs4812325 | 20 | 37485458 | G | A | 0.370 | -0.072 | 0.009 | 8.96E-16 | 65.27 |
| SCZ | rs5751191 | 22 | 42370991 | T | C | 0.484 | -0.066 | 0.009 | 3.00E-14 | 58.18 |
| SCZ | rs58120505 | 7 | 2029867 | T | C | 0.602 | 0.090 | 0.009 | 2.24E-24 | 103.7 |
| SCZ | rs61937595 | 12 | 57682956 | C | T | 0.917 | 0.130 | 0.016 | 1.15E-15 | 64.49 |
| SCZ | rs6482437 | 10 | 18726326 | A | C | 0.099 | -0.099 | 0.014 | 3.33E-12 | 48.51 |
| SCZ | rs6943762 | 7 | 86403263 | T | C | 0.883 | 0.105 | 0.013 | 1.57E-15 | 63.39 |
| SCZ | rs72802868 | 5 | 152235215 | G | T | 0.724 | 0.069 | 0.010 | 4.55E-13 | 51.96 |
| SCZ | rs7647398 | 3 | 180733150 | C | T | 0.811 | 0.077 | 0.011 | 1.07E-12 | 50.55 |
| SCZ | rs9318627 | 13 | 79930079 | A | C | 0.613 | 0.061 | 0.009 | 4.35E-12 | 48.36 |
| SCZ | rs9636107 | 18 | 53200117 | A | G | 0.506 | -0.070 | 0.009 | 5.12E-16 | 66.06 |

ADHD, attention-deficit/hyperactivity disorder; AN, Anorexia nervosa; ANX, anxiety disorder; ASD, autism spectrum disorder; BD, bipolar disorder; CHR, chromosome; EAF, effect allele frequency; MDD, major depressive disorder; OCD, obsessive-compulsive disorder; PTSD, post-traumatic stress disorder; SCZ, schizophrenia; SE, standard error; SNP, single nucleotide polymorphism.
