# Supplementary material for: Obstructive sleep apnea and mental disorders: a bidirectional mendelian randomization study
Source: BMC Psychiatry. 2024 Apr 23;24:304. doi: 10.1186/s12888-024-05754-8 (PMC11040841; doi:10.1186/s12888-024-05754-8)
Supplement: Supplementary file 4 — Supplementary Material 4 [file 12888_2024_5754_MOESM4_ESM.doc]

**Additional file 4. Associations between genetic liability for obstructive sleep apnea and risk of mental disorders.**

| **Exposure** | **Outcome** | **Method** | **Used SNPs** | **Beta** | **SE** | **OR (95% CI)** | ***P*-value** | **Q *P*-value** | ***P_intercept_*-value** | **Statistical power(%)** |
| --- | --- | --- | --- | --- | --- | --- | --- | --- | --- | --- |
| OSA | ADHD | IVW | 8 | 0.232 | 0.108 | 1.26 (1.02-1.56) | 0.032 | 0.150 |  | 25 |
|  |  | MR Egger | 8 | 0.206 | 0.446 | 1.23 (0.51-2.95) | 0.661 | 0.097 | 0.954 |  |
|  |  | Weighted median | 8 | 0.158 | 0.119 | 1.17 (0.93-1.48) | 0.184 |  |  |  |
| OSA | AN | IVW | 7 | 0.049 | 0.138 | 1.05 (0.80-1.37) | 0.724 | 0.584 |  | 5 |
|  |  | MR Egger | 7 | 0.158 | 0.546 | 1.17 (0.40-3.42) | 0.783 | 0.460 | 0.843 |  |
|  |  | Weighted median | 7 | 0.052 | 0.190 | 1.05 (0.73-1.53) | 0.783 |  |  |  |
| OSA | ANX | IVW | 6 | -0.190 | 0.327 | 0.83 (0.44-1.58) | 0.571 | 0.297 |  | 6 |
|  |  | MR Egger | 6 | -0.200 | 1.754 | 0.82 (0.03-25.54) | 0.916 | 0.192 | 0.995 |  |
|  |  | Weighted median | 6 | 0.057 | 0.374 | 1.06 (0.51-2.20) | 0.878 |  |  |  |
| OSA | ASD | IVW | 8 | 0.141 | 0.131 | 1.15 (0.89-1.49) | 0.283 | 0.841 |  | 7 |
|  |  | MR Egger | 8 | 0.195 | 0.505 | 1.22 (0.45-3.27) | 0.713 | 0.753 | 0.915 |  |
|  |  | Weighted median | 8 | 0.196 | 0.165 | 1.22 (0.88-1.68) | 0.235 |  |  |  |
| OSA | BD | IVW | 8 | -0.010 | 0.086 | 0.99 (0.84-1.18) | 0.943 | 0.670 |  | 5 |
|  |  | MR Egger | 8 | -0.04 | 0.322 | 0.96 (0.51-1.81) | 0.907 | 0.556 | 0.918 |  |
|  |  | Weighted median | 8 | 0.021 | 0.108 | 1.02 (0.83-1.26) | 0.849 |  |  |  |
| OSA | MDD | IVW | 7 | 0.049 | 0.049 | 1.05 (0.96-1.16) | 0.310 | 0.198 |  | 8 |
|  |  | MR Egger | 7 | 0.102 | 0.205 | 1.11 (0.74-1.66) | 0.640 | 0.132 | 0.801 |  |
|  |  | Weighted median | 7 | 0.054 | 0.056 | 1.06 (0.95-1.18) | 0.335 |  |  |  |
| OSA | OCD | IVW | 8 | 0.450 | 0.406 | 1.57 (0.71-3.47) | 0.268 | 0.133 |  | 10 |
|  |  | MR Egger | 8 | 2.511 | 1.431 | 12.31 (0.75-203.40) | 0.130 | 0.229 | 0.186 |  |
|  |  | Weighted median | 8 | 0.707 | 0.496 | 2.03 (0.77-5.36) | 0.154 |  |  |  |
| OSA | PTSD | IVW | 8 | 0.103 | 0.172 | 1.11 (0.79-1.55) | 0.551 | 0.146 |  | 7 |
|  |  | MR Egger | 8 | 0.64 | 0.678 | 1.90 (0.50-7.16) | 0.382 | 0.136 | 0.443 |  |
|  |  | Weighted median | 8 | 0.173 | 0.200 | 1.19 (0.80-1.76) | 0.386 |  |  |  |
| OSA | SCZ ^a^ | IVW | 7 | 0.125 | 0.096 | 1.13 (0.94-1.37) | 0.193 | 0.259 |  | 9 |
|  |  | MR Egger | 7 | 0.440 | 0.366 | 1.55 (0.77-3.18) | 0.284 | 0.246 | 0.412 |  |
|  |  | Weighted median | 7 | 0.193 | 0.114 | 1.21 (0.97-1.52) | 0.091 |  |  |  |

ADHD, attention-deficit/hyperactivity disorder; AN, Anorexia nervosa; ANX, anxiety disorder; ASD, autism spectrum disorder; BD, bipolar disorder; IVW, inverse-variance weighted; MDD, major depressive disorder; OCD, obsessive-compulsive disorder; OR (95% CI), odds ratio for the outcome and 95% confidence interval of odds ratio estimate; OSA, obstructive sleep apnea; *P_intercept_* -value, the *P* value for MR-Egger intercept; PTSD, post-traumatic stress disorder; Q *P*-value, P-value for Cochran's Q statistic (IVW) and Rucker's Q statistic (MR Egger); SCZ, schizophrenia; SE, standard error; SNP, single nucleotide polymorphism.

^a^ For outcome phenotype SCZ, the table shows the results of the second MR analysis with outlier SNPs removed due to heterogeneity.
