# Supplementary material for: Obstructive sleep apnea and mental disorders: a bidirectional mendelian randomization study
Source: BMC Psychiatry. 2024 Apr 23;24:304. doi: 10.1186/s12888-024-05754-8 (PMC11040841; doi:10.1186/s12888-024-05754-8)
Supplement: Supplementary file 5 — Supplementary Material 5 [file 12888_2024_5754_MOESM5_ESM.doc]

**Additional file 5. Associations between genetic liability for mental disorders and risk of obstructive sleep apnea.**

| **Exposure** | **Outcome** | **Method** | **Used SNPs** | **Beta** | **SE** | **OR (95% CI)** | ***P*-value** | **Q *P*-value** | ***P_intercept_*-value** | **Statistical power(%)** |
| --- | --- | --- | --- | --- | --- | --- | --- | --- | --- | --- |
| ADHD ^a^ | OSA | IVW | 9 | 0.060 | 0.056 | 1.06 (0.95-1.18) | 0.282 | 0.100 |  | 7 |
|  |  | MR Egger | 9 | 0.118 | 0.150 | 1.13 (0.84-1.51) | 0.456 | 0.071 | 0.684 |  |
|  |  | Weighted median | 9 | 0.063 | 0.066 | 1.06 (0.94-1.21) | 0.344 |  |  |  |
| AN | OSA | IVW | 7 | 0.001 | 0.039 | 1.00 (0.93-1.08) | 0.973 | 0.837 |  | 5 |
|  |  | MR Egger | 7 | 0.201 | 0.322 | 1.22 (0.65-2.30) | 0.559 | 0.795 | 0.559 |  |
|  |  | Weighted median | 7 | 0.010 | 0.052 | 1.01 (0.91-1.12) | 0.842 |  |  |  |
| ANX | OSA | IVW | 6 | 0.016 | 0.019 | 1.02 (0.98-1.06) | 0.397 | 0.482 |  | 6 |
|  |  | MR Egger | 6 | -0.023 | 0.058 | 0.98 (0.87-1.09) | 0.709 | 0.411 | 0.508 |  |
|  |  | Weighted median | 6 | -0.009 | 0.026 | 0.99 (0.94-1.04) | 0.715 |  |  |  |
| ASD ^b^ | OSA | IVW | 19 | 0.002 | 0.027 | 1.00 (0.95-1.06) | 0.947 | 0.354 |  | 5 |
|  |  | MR Egger | 19 | 0.098 | 0.080 | 1.10 (0.94-1.29) | 0.237 | 0.395 | 0.219 |  |
|  |  | Weighted median | 19 | -0.004 | 0.035 | 1.00 (0.93-1.07) | 0.912 |  |  |  |
| BD ^a^ | OSA | IVW | 25 | 0.035 | 0.030 | 1.04 (0.98-1.10) | 0.238 | 0.211 |  | 6 |
|  |  | MR Egger | 25 | -0.036 | 0.160 | 0.96 (0.70-1.32) | 0.823 | 0.181 | 0.654 |  |
|  |  | Weighted median | 25 | -0.007 | 0.038 | 0.99 (0.92-1.07) | 0.844 |  |  |  |
| MDD ^a^ | OSA | IVW | 23 | 0.316 | 0.069 | 1.37 (1.20-1.57) | <0.001 | 0.132 |  | 60 |
|  |  | MR Egger | 23 | 0.224 | 0.533 | 1.25 (0.44-3.55) | 0.678 | 0.104 | 0.864 |  |
|  |  | Weighted median | 23 | 0.212 | 0.092 | 1.24 (1.03-1.48) | 0.021 |  |  |  |
| OCD | OSA | IVW | 9 | 0.003 | 0.016 | 1.00 (0.97-1.04) | 0.831 | 0.248 |  | 5 |
|  |  | MR Egger | 9 | 0.018 | 0.048 | 1.02 (0.93-1.12) | 0.715 | 0.184 | 0.750 |  |
|  |  | Weighted median | 9 | 0.012 | 0.020 | 1.01 (0.97-1.05) | 0.554 |  |  |  |
| PTSD ^a^ | OSA | IVW | 13 | 0.011 | 0.033 | 1.01 (0.95-1.08) | 0.736 | 0.077 |  | 5 |
|  |  | MR Egger | 13 | 0.096 | 0.063 | 1.10 (0.97-1.24) | 0.156 | 0.140 | 0.150 |  |
|  |  | Weighted median | 13 | -0.025 | 0.041 | 0.98 (0.90-1.06) | 0.545 |  |  |  |
| SCZ ^a^ | OSA | IVW | 20 | 0.012 | 0.028 | 1.01 (0.96-1.07) | 0.673 | 0.204 |  | 5 |
|  |  | MR Egger | 20 | -0.130 | 0.154 | 0.88 (0.65-1.19) | 0.411 | 0.202 | 0.362 |  |
|  |  | Weighted median | 20 | -0.013 | 0.038 | 0.99 (0.92-1.06) | 0.734 |  |  |  |

ADHD, attention-deficit/hyperactivity disorder; AN, Anorexia nervosa; ANX, anxiety disorder; ASD, autism spectrum disorder; BD, bipolar disorder; IVW, inverse-variance weighted; MDD, major depressive disorder; OCD, obsessive-compulsive disorder; OR (95% CI), odds ratio for the outcome and 95% confidence interval of odds ratio estimate; OSA, obstructive sleep apnea; *P_intercept_* -value, the *P* value for MR-Egger intercept; PTSD, post-traumatic stress disorder; Q *P*-value, P-value for Cochran's Q statistic (IVW) and Rucker's Q statistic (MR Egger); SCZ, schizophrenia; SE, standard error; SNP, single nucleotide polymorphism.

^a^ For outcome phenotype ADHD, BD, MDD, PTSD and SCZ, the table shows the results of the second MR analysis with outlier SNPs removed due to heterogeneity.

^b^ For exposure phenotype ASD, all results are shown after the removal of outliers with the MR-PRESSO test.
