# Supplementary material for: Obstructive sleep apnea and mental disorders: a bidirectional mendelian randomization study
Source: BMC Psychiatry. 2024 Apr 23;24:304. doi: 10.1186/s12888-024-05754-8 (PMC11040841; doi:10.1186/s12888-024-05754-8)
Supplement: Supplementary file 6 — Supplementary Material 6 [file 12888_2024_5754_MOESM6_ESM.doc]

**Additional file 6. MR analysis plot of the association between genetic liability for major depressive disorder and risk of obstructive sleep apnea.**


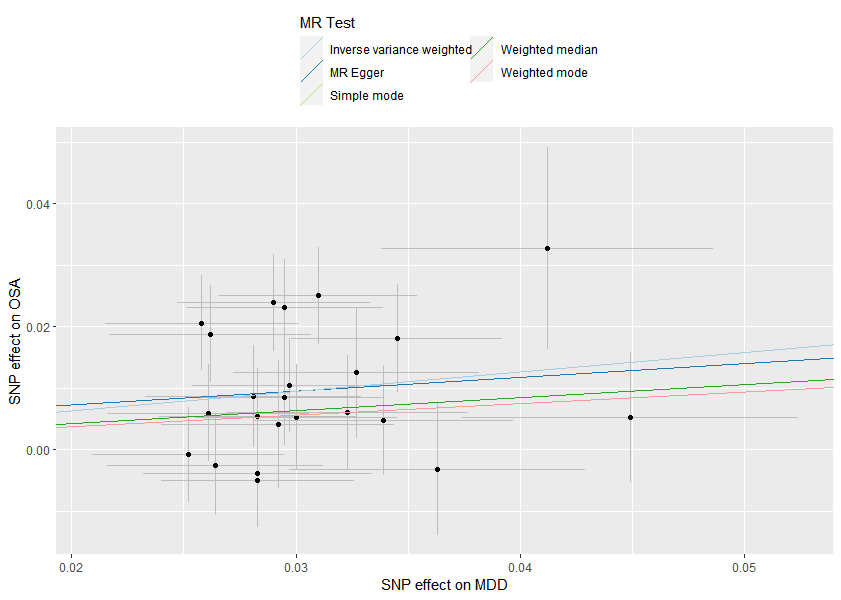


Figure 1. Scatterplot of Associations of Genetic Risk of MDD on Risk of OSA.

MDD, major depressive disorder; OSA, obstructive sleep apnea; SNP, single nucleotide polymorphism.


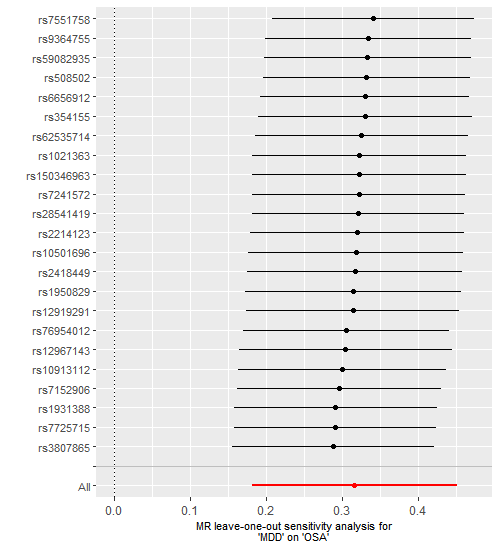


Figure 2. The leave-one-out-sensitivity forest plot of Associations of Genetic Risk of MDD on Risk of OSA.

MDD, major depressive disorder; OSA, obstructive sleep apnea.


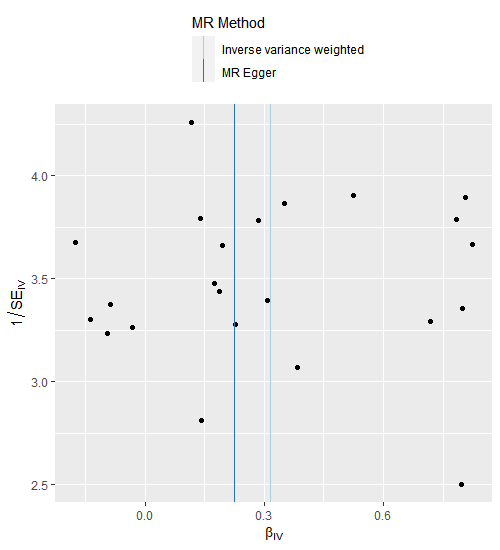


Figure 3. Funnel plot of Associations of Genetic Risk of MDD on Risk of OSA.
